# Oral microbiome associated with differential ratios of Porphyromonas gingivalis and Streptococcus cristatus

**DOI:** 10.21203/rs.3.rs-3266326/v1

**Published:** 2023-08-22

**Authors:** Qingguo Wang, Bing-Yan Wang, Siddharth Pratap, Hua Xie

**Affiliations:** Meharry Medical College; University of Texas Health Science Center at Houston; Meharry Medical College; Meharry Medical College

**Keywords:** Porphyromonas gingivalis, periodontitis, oral microbiome, shotgun metagenomic sequencing

## Abstract

**Background:**

Periodontitis has been recently defined as a dysbiotic disease resulting from imbalanced oral microbiota. The transition of microbial communities from commensal to periodontitis-associated ones likely requires colonization by specific pathogens, including *Porphyromonas gingivalis*. We previously reported an antagonistic relationship between *Streptococcus cristatus* and *P. gingivalis* and the role of *S. cristatus* in inhibition of the biofilm formation, invasion, and gingipain enzymatic activity of *P. gingivalis*. Given the importance of *P. gingivalis* as a keystone pathogen of polymicrobial communities, the determinants of *P. gingivalis* levels, its interaction with the core microbiota, and association with the pathogenic potential of the microbial communities need to be addressed.

**Results:**

This present study intends to determine the role of *S. cristatus* in altering interactions of *P. gingivalis* with other oral bacteria in a complex context. We collected dental plaque samples from periodontitis patients and assigned them into two groups based on their ratios of *S. cristatus* and *P. gingivalis*. We then characterized microbial profiles of the dental plaque samples using shotgun metagenomic sequencing and subsequently compared oral microbial composition and functional capabilities between groups with high or low *S. cristatus-P. gingivalis* ratios. Taxonomic annotation showed significant differences in microbial compositions at both genus and species levels between the two groups. Notably, a higher microbial composition diversity was observed in the samples with low *S. cristatus-P. gingivalis* ratios. The antibiotic resistance gene profiles of the two groups are also distinct, with significantly increased diversity and abundance of antibiotic resistance genes in the dental plaque samples with low *S. cristatus-P. gingivalis* ratios, which likely lead to elevated virulence potential.

**Conclusions:**

Overall, our work highlights the importance of *S. cristatus-P. gingivalis* ratios in influencing the virulence of the oral microbiome. Approaches to enhance *S. cristatus-P. gingivalis* ratios in oral microbial communities will be attractive for revising the dysbiotic oral microbiome.

## Introduction

Periodontitis is one of the most common diseases in humans, affecting ~ 42% of adults aged 30 years and older in the US. The prevalence of periodontitis is influenced by multiple factors including age, gender, socioeconomic status, and race/ethnicity (1). The oral microbiome includes hundreds of bacterial species and phylotypes and is considered an important contributor to the development of periodontitis (2, 3). Understanding the effects of individual bacterial species and their relationship with others in complex oral microbial communities is crucial for the identification of microbiological factors associated with susceptibility to and severity of periodontitis.

*Porphyromonas gingivalis* is known to play a vital role in the development of dysbiotic microbial communities and can disrupt host-microbial homeostasis and induce inflammatory responses by acting with other oral bacteria. Therefore, it is considered a keystone-pathogen of periodontitis (4, 5). We previously reported an antagonistic relationship between *Streptococcus cristatus* and *P. gingivalis* and identified *S. cristatus* ArcA and a streptococcal ArcA derived anti-P. *gingivalis* peptide (SAPP) that can effectively inhibit biofilm formation, invasion, and gingipain enzymatic activity of *P. gingivalis* (6-10). We also demonstrated using a mouse model that SAPP can reduce *P. gingivalis*-induced alveolar bone loss (11, 12) and showed using *ex-vivo* assays that it can eliminate *P. gingivalis* and other periodontitis-associated bacteria from the oral microbial communities (11, 13). Our clinical studies showed a negative correlation between the distributions of *S. cristatus* and *P. gingivalis* in dental plaques derived from periodontitis patients and a higher *S. cristatus-P. gingivalis* ratios in dental plaque samples collected from Caucasian Americans (CAs) periodontitis patients, compared to those from African Americans (AAs) and Hispanic Americans (HAs) patients (14, 15). However, it is unclear whether different *S. cristatus-P. gingivalis* ratios accompany particular oral microbial profiles and functions, and if *S. cristatus-P. gingivalis* ratios can be used as clinical parameters for predicting periodontitis progression.

The goal of this work is to identify similarities and differences shared among dental samples with relatively high or low *S. cristatus-P. gingivalis* ratios. Our overall hypothesis is that the ratio of *S. cristatus-P. gingivalis* may play a vital role in the pathogenic properties of the oral microbiome and contribute to the pathogenetic potential of the oral microbiota. Previously, using qPCR, levels of *P. gingivalis, S. cristatus*, and total bacteria in the dental plaque from periodontitis patients were identified, and *S. cristatus-P. gingivalis* ratio of each sample was calculated (14). In this study, we profiled microbial communities at the species level and characterized the functional and biological process of microbial communities using metagenome shotgun sequencing. By comparing abundance and diversity of microbial taxa in the dental plaque samples with relatively high *S. cristatus-P. gingivalis* ratios to those with the low ratios, our approach identified differential composition and abundance of oral microbial communities with different *S. cristatus-P. gingivalis* ratios. Additionally, we found increased diversity and abundance of antibiotic resistance genes in the samples with low ratios. Altogether, the comparative study revealed an involvement of *S. cristatus* and *P. gingivalis* ratios in regulating the pathogenicity of oral microbial communities.

## Methods

### Study cohorts.

The research protocol was approved by the Committee for the Protection of Human Subjects of the University of Texas Health Science Center at Houston (IRB number: HSC-DB-17-0636). Candidates were screened during routine dental visits in clinic at the School of Dentistry, University of Texas Health Science Center at Houston between 2017 and 2022. Individuals aged 21 –75 with self-reported ethnicity/race of AAs, CAs, or HAs were enrolled after the initial periodontal examination that included determination of plaque index (PI) and bleeding on probing (BOP) (16). Radiographs were taken during this screening phase to assess bone loss. The clinical oral examinations were performed by trained dental examiners who are faculty members of the School of Dentistry, University of Texas Health Science Center at Houston. The examiners are calibrated in diagnosis of periodontitis annually. All study participants were diagnosed with generalized periodontitis Stage II or III, regardless of their grading, based on the 2017 World Workshop classification (17, 18). The enrolled patients also met the following criteria: ≤4 tooth loss due to periodontitis, interdental CAL ≥ 3mm and PD ≥ 5 mm at two or more teeth in different quadrants, and radiographic bone loss ≥ 15%. Other criteria for study participation were 1) no scaling and root planning within the previous year or periodontal surgeries in the previous five years; 2) no antibiotic therapy in the previous six months; 3) not pregnant. Information on demographics and self-reported dental and medical histories of the participants were abstracted from the Electronic Health Record.

### Dental plaque sample collection.

Dental plaque samples were collected by board certified periodontists using sterile paper points at baseline prior to any dental treatment and labelled with numbers according to sampling sequences. The paper points were placed in ≥ 5mm pockets in different quadrants for 1 minute and then immersed immediately in an Eppendorf tube with 0.5 ml of Tris-EDTA (TE) buffer (pH 7.5) (15). Oral bacteria were harvested by centrifugation and bacterial pellets were resuspended in 100 μl TE buffer.

### Sequencing and quality control.

The samples were sent to Novogene Co. (Sacramento, CA) for metagenomic sequencing. Briefly, DNA extracted from human dental plaques was randomly sheared into short fragments. The obtained fragments were end repaired, A-tailed and further ligated with Illumina adapters. The fragments with adapters were then PCR amplified, size selected, and purified. Quality control of the library was conducted with Qubit (≥ 20 ng, ≥ 10ng/μl) and real-time PCR for quantification and bioanalyzer for size distribution detection. Quantified libraries were pooled and sequenced on an Illumina NovaSeq high throughput sequencer by Novogene Corporation, Inc., with paired-end sequencing length of 150bp and an output of ~ 6GB raw data per sample.

### Data preprocessing.

The amount of raw data generated per sample is 6.276GB on average. To ensure accuracy and reliability of the subsequent data analysis, we trimmed all low-quality bases. We also discarded the reads which contain uncalled N nucleotide stretching over 10bp and reads which overlap with adapter for more than 15 bp. To minimalize host DNA contamination, we also discarded the raw reads mapping to the human reference genome using Bowtie2 (19). After quality control and host-exclusion filtering, the amount of clean data was reduced slightly to 6.275GB per sample, with 96.66% and 92.37% bases having PHRED quality scores greater than 20 and 30, respectively.

### Gene prediction and abundance analysis.

Software MetaGeneMark (version 2.10) was used to predict Open Reading Frames (ORFs) from Scaftigs (≥ = 500bp) (20, 21). ORFs less than 100nt were discarded. The ORFs were then dereplicated using CD-HIT (22, 23) which was run in default settings (identity = 95%, coverage = 90%) to generate gene catalogues. To calculate gene quantity, clean raw data were mapped to the gene catalogue using Bowtie2 (parameters: -end-to-end, -sensitive, -I 200, -X 400). Gene abundance $G_{k}$ was calculated based on the total number of mapped reads ‘‘r" and gene length ‘‘L", using the formula as follows:

$$G_{k}=\frac{r_{k}}{L_{k}}\frac{1}{\sum_{i=1}^{n}\frac{r_{i}}{L_{i}}}$$

where r stands for number of mapping reads and L stands for the length of gene. Downstream analyses were performed based on the abundance of gene catalogues.

### Statistical analysis.

Statistical analyses were performed using scipy.stats in SciPy (version 1.4.1), an open-source Python library for scientific computing. Independent two sample *t*-tests were performed to compare the samples with high *S. cristatus* and *P. gingivalis* ratios to those with low ratios. The *t*-tests were two-sided, and an assumption of identical variances on the sample distributions was used. The threshold of statistical significance was set to p-value ≤ 0.05.

## Results

### Characteristics of the study cohort.

We previously investigated the oral microbial profiles of dental plaques derived from periodontitis patients with different racial/ethnic backgrounds and using qPCR measured the distribution and levels of several well-studied oral bacteria in their dental plaque samples. These included keystone pathogens, accessory pathogens, and pathobionts (24). To examine the role of *P. gingivalis* and its antagonistic species *S. cristatus* in the composition of the oral microbiota, we selected 14 samples with relatively low *S. cristatus-P. gingivalis* ratios (< 1) and 16 samples with the higher ratios (> 100) for metagenome shotgun sequencing. The general characteristics of the participants included in the present study is shown in Table 1. Significant differences in gender, age, BOP, PI, and number of teeth between the two groups were not observed (Table 1).

### Diversity and similarity of oral microbiota with different S. cristatus-P. gingivalis ratios.

**Figure 1:**
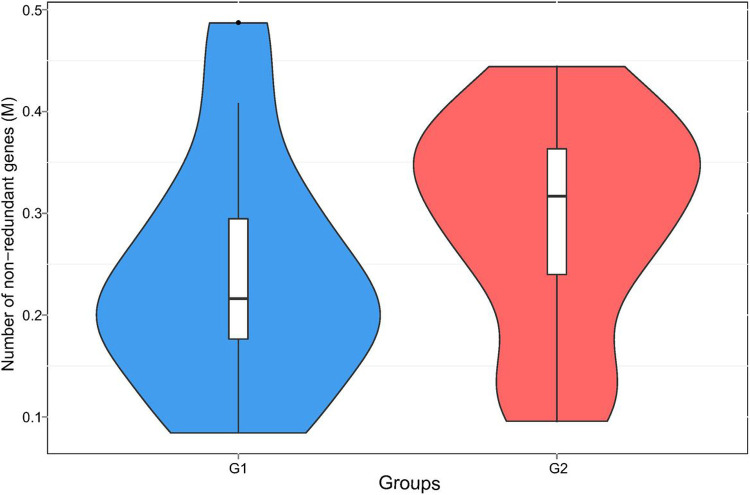
Comparison of non-redundant genes between samples with different *S. cristatus-P. gingivalis* ratios. The gene abundance was calculated based on the total number of mapped reads and gene lengths. Blue violin represents richness and abundance of non-redundant genes in the samples with lower *S. cristatus-P. gingivalis*ratios (G1). Red violin represents richness and abundance of non-redundant genes in the samples with higher *S. cristatus-P. gingivalis*ratios (G2).

**Figure 2:**
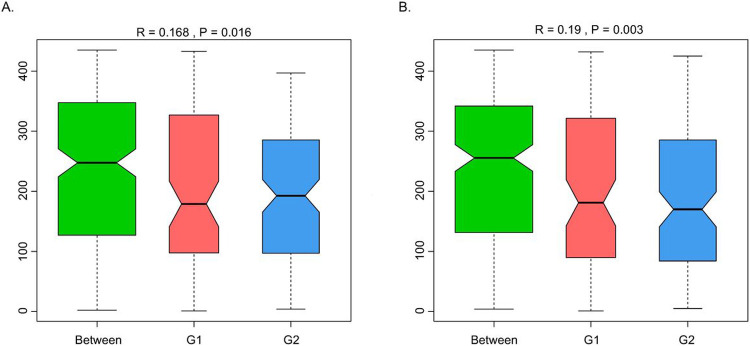
Analysis of dissimilarities between the groups with different *S. cristatus-P. gingivalis* ratios. ANOSIM test was used to compare the mean of ranked dissimilarities between group of samples with lower *S. cristatus-P. gingivalis*ratios (G1) and group with a higher ratio (G2) at (A) genus level and (B) species level. The red and blue boxes stand for dissimilarities between samples within each group, whilst green box (Between) represents dissimilarities of pairs of samples from two different groups. Positive R values 0.168 and 0.19 and p-value<0.05 indicate that inter-group variation is statistically more significant than intra-group variation.

**Figure 3:**
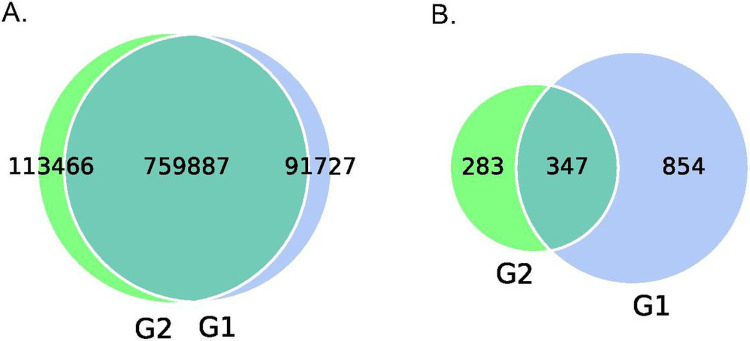
Differences in genes and taxonomy between groups. (A) Total genes annotated in groups with low (G1) or high (G2) *S. cristatus-P. gingivalis*ratios are presented in a Venn figure. Green and blue areas represent number of peculiar genes identified within each group, respectively, and an overlap area represents the number of common genes found in both groups. (B) Taxonomic difference between the groups at the species level.

A total of 965,080 genes were identified using MetaGeneMark (20, 21). Figure 1 shows the mean and median number of non-redundant genes (238,472 and 216,072, respectively) in samples with lower *S. cristatus-P. gingivalis* ratio (< 1). While the mean and median of gene counts is 294,355 and 316,927, respectively, in samples with higher *S. cristatus-P. gingivalis* ratio (> 100). Although a statistically significant difference in numbers of the non-redundant genes was not reached between the groups with different *S. cristatus-P. gingivalis* ratios, significant abundance dissimilarities were found at bacterial genus and species levels between these groups using ANOSIM analysis, a non-parametric test based on ranked dissimilarity measure (Fig. 2). This suggests a higher similarity of gene abundance within each group than the similarity between the groups at the genus level (R = 0.168, *p* = 0.016) and the species level (R = 0.19, *p* = 0.003). Moreover, non-redundant genes were observed in both the groups as shown in Venn diagrams (Fig. 3A). Among the 965,080 non-redundant genes identified, 91,727 unique genes (9.50%) were detected in the group with low *S. cristatus-P. gingivalis* ratios (G1), while 113,466 (11.75%) were observed in the group with the high ratios (G2). Further, taxonomic annotations identified a total of 1484 microbial species in the cohort. Microbial taxa were found more diverse in the samples with low *S. cristatus-P. gingivalis* ratios. A total of 1201 species were identified in the group with low *S. cristatus-P. gingivalis* ratios, while only 630 species in the group with high *S. cristatus-P. gingivalis* ratios. Among the 1201 microbial taxa annotated in the group with low *S. cristatus-P. gingivalis* ratios, 854 group-specific species (58.6%) were found compared to 283 unique species (19.1 %) in the group with the high ratios. A total of 347 species annotated in both groups. These results indicate a more complex profile of microbial species in the group with low *S. cristatus-P. gingivalis* ratios (Fig. 3B).

**Figure 4:**
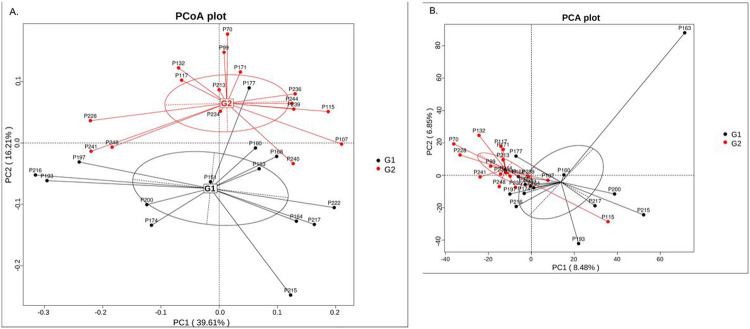
Schematic of PCoA (A), PCA(B), and NMDS (C) plots. Data were analyzed at bacterial species level. Black dots represent samples with lower ratio of *S. cristatus-P. gingivalis*, and red ones denote samples with the higher ratios. In plots (A) and (C), the distance between each pair of samples represents the dissimilarity between them.

We also conducted Principal Coordinates Analysis (PCoA), Principal Component Analysis (PCA), and Non-metric MultiDimensional Scaling (NMDS) analysis, an indirect gradient analysis approach that produces an ordination based on a distance matrix. Figure 4 provides the visualization of the results of PCoA, PCA, and NMDS analysis. In Fig. 4A and 4C, the distance between each pair of samples represents the dissimilarity between them. These two plots show clear distinction between the two groups with different *S. cristatus-P. gingivalis* ratios (G1 and G2), with samples within each group gathering closer together. The PCA plot in Fig. 4B also shows the overall separation between the two groups.

### Abundance of common bacterial taxa.

**Figure 5:**
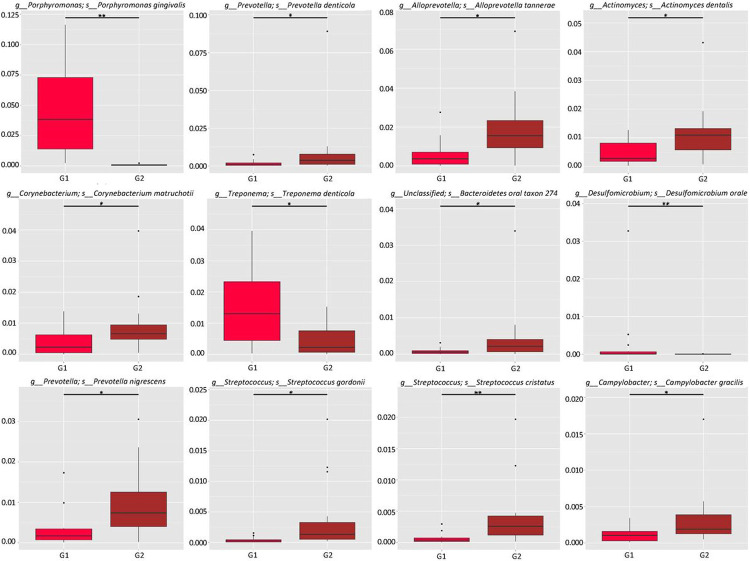
Relative abundance of the top 12 bacterial species. The x and y axis of boxplots represent bacterial abundance and sample groups, respectively. The oral microbial richness and evenness were estimated using Metastats analysis, and the differences between groups with low (G1) or high (G2) ratios of *S. cristatus-P. gingivalis* were determined using a non-parametric *t*-test. Asterisk ‘*’ means *P* value between the two groups is smaller than 0.05, and double asterisks ‘**’ means *P* value is smaller than 0.01.

**Figure 6:**
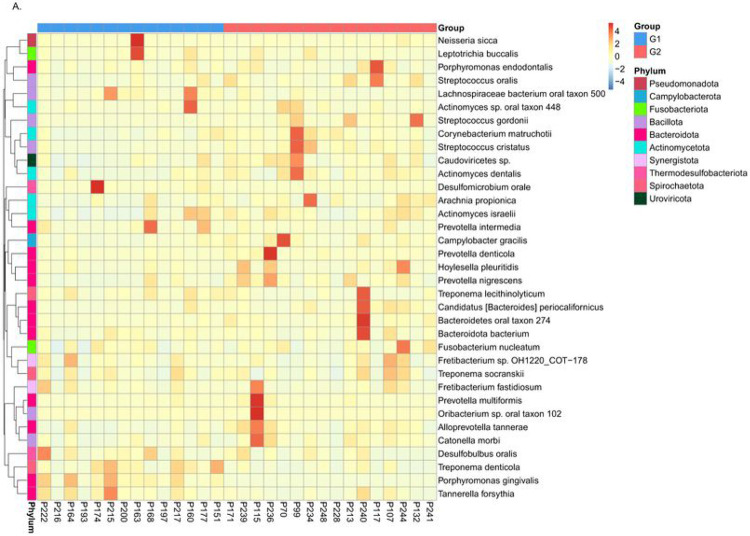
Abundance heatmap based on the top 35 species derived from metagenomic sequencing data. (A) A heatmap of all 30 samples. X-axis represents the sample information; Y-axis represents species annotation; and the left side of heatmap is clustering tree of species. The value of the heatmap is standardized Z score. (B) A heatmap by groups.

Metastats analysis (25) was performed to detect differential abundance of common oral bacteria between two groups with different *S. cristatus-P. gingivalis* ratios. As shown in Fig. 5, three bacterial species, *P. gingivalis, Treponema denticola*, and *Desulfomicrobium orale*, out of the 12 most abundant taxa, were detected 56.08, 5.83, and 5.62-fold more abundant, respectively, in samples with low *S. cristatus-P. gingivalis* ratios, compared to the samples with high ratios. The other nine common bacterial taxa (*Prevotella denticola, Alloprevotella tannerae, Actinomyces dentalis, Corynebacterium matruchotii, Bacteroidetes oral taxon 274, Prevotella nigrescens, Streptococcus gordonii, S. cristatus*, and *Campylobacter gracilis*) were 1.80-fold to 6.77-fold more abundant in samples with the high ratios than those with the low ratios. In addition, the top 35 microbial taxa with differential abundance between groups are shown in an abundance heatmap (Fig. 6A). Among them, eleven species exhibit a higher abundance in the group with lower *S. cristatus-P. gingivalis* ratio, compared to the group with a higher ratio (Fig. 6A and B). These observations are consistent with previous studies of microbial profiles of dental plaques (26, 27), where a relatively higher abundance of *P. gingivalis* was found with elevated levels of *T. denticola, Tannerella forsythia, Filifacter alocis* in the group with low *S. cristatus-P. gingivalis* ratios, compared to those found in the group with high ratios. Other bacterial species found more abundance in the group with low ratios were *Treponema lecithinolyticum, Treponema maltophilum, Treponema vincentii, Desulfobulbus oralis, Fretibacterium sp.* OH1220_COT-178, *Prevotella intermedia*, and *Lachnospiraeae bacterium* oral taxon 500 (Fig. 6B). In contrast, species of *Streptococcus* and *Actinomyces* were dominated in the samples with the higher ratios, in which more than six-fold higher level of *S. cristatus* was discovered. Notably, hierarchical clustering algorithms showed that along with *T. denticola, T. forsythia*, another bacterial species, *D. oralis*, was often positively co-occurrent in the samples (Fig. 6A). While, *S. cristatus* was clustered with *C. matruchotii, Actinomyces dentalis*, and *S. gordonii*. Interestingly, *S. cristatus* was clustered with *Caudoviricetes sp.*, bacteriophages with dsDNA genomes, which are highly prevalent in human gastrointestinal tract (28). These results revealed differential levels of microbial taxa in the samples with low or high *S. cristatus-P. gingivalis* ratios, respectively, suggesting that different core microbiotas are configured based on the *S. cristatus-P. gingivalis* ratios.

### Functional profile of oral microbiome with different S. cristatus-P. gingivalis ratios.

**Figure 7:**
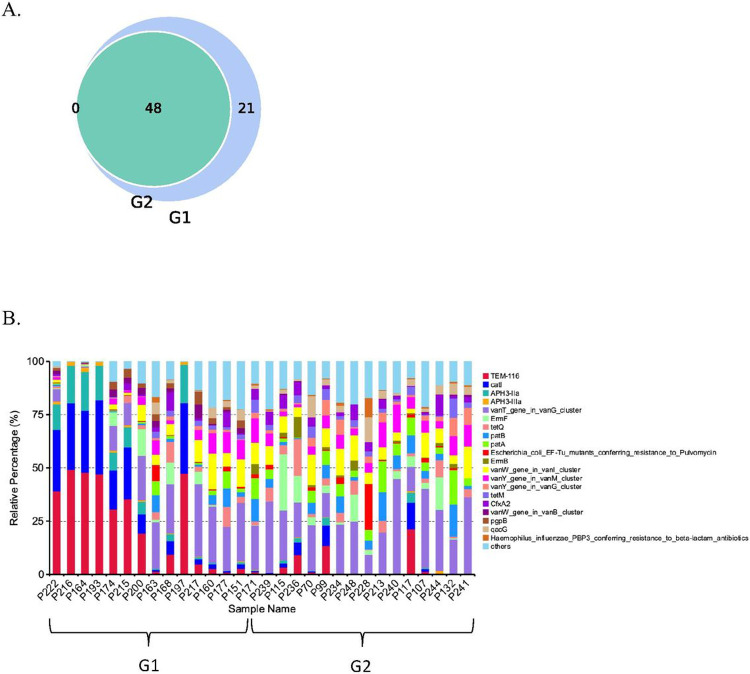
Identification of antibiotic resistance genes in the dental plaque samples. (A) The total number of antibiotic resistant genes identified in 30 samples was presented in a Venn diagram. Blue circle represents number of antibiotic resistant genes were detected in the group with low ratios, and green circle shows the genes in the group with high *S. cristatus-P. gingivalis* ratios. The unique genes are in area that is not overlapped. (B) Each stacked bar represents relatively abundance of antibiotic resistance genes in each sample.

To determine functional diversity of bacterial communities with lower and higher *S. cristatus-P. gingivalis* ratios, we mapped function annotation against several functional databases such as Comprehensive Antibiotic Resistance Database (CARD) (29), Carbohydrate-Active enzymes Database (CAZy) (30), Kyoto Encyclopedia of Genes and Genomes (KEGG) (31), and Genealogy of Genes: Non-supervised Orthologous Groups (eggNOG) (32). As shown in Fig. 7A, sixty-nine antibiotic resistance genes were identified in the 30 dental plaque samples, with forty-eight of them found in all samples at different abundance levels. Twenty-one antibiotic resistant genes, such as multidrug efflux pump membrane fusion proteins (mdtA, B, F, and G), were unique to the group with low *S. cristatus-P. gingivalis* ratios, although with relatively low abundance. Percentage of antibiotic resistance genes in each sample was indicated in Fig. 7B, and absolute copies of antibiotic resistance genes were listed in Table 2. Among the top 30 antibiotic resistance genes annotated, six were significantly abundant in samples with lower *S. cristatus-P. gingivalis* ratios compared to those with higher ratios, while seven genes were more prevalent in the samples with higher ratios. The antibiotic resistance genes that exhibit remarkably differential abundance between two groups include *Tem*-116, *catI, aph3-IIa*, and *aph3-IIIa* with increased levels in the samples with the low *S. cristatus-P. gingivalis* ratios (Table 2). These results of differential richness of antibiotic resistance genes in the two groups indicate a potential regulatory mechanism contributing to the physiology of the oral microbiome.

**Figure 8:**
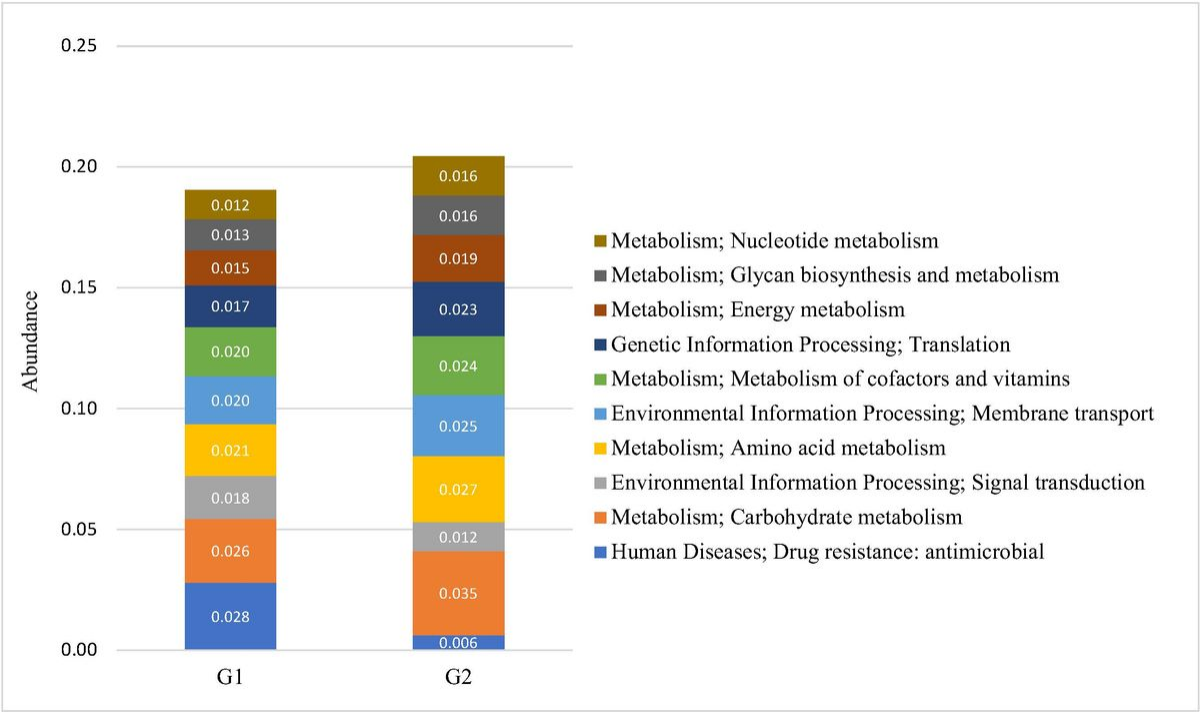
The functional diversities of groups with low (G1) or high (G2) *S. cristatus-P. gingivalis* ratios. Metagenomic proteins were quantified by annotating metagenomic sequences with functions. Protein coding sequences were mapped against functional databases. Stacked bar represents proportion of relative functional abundance.

**Figure 9:**
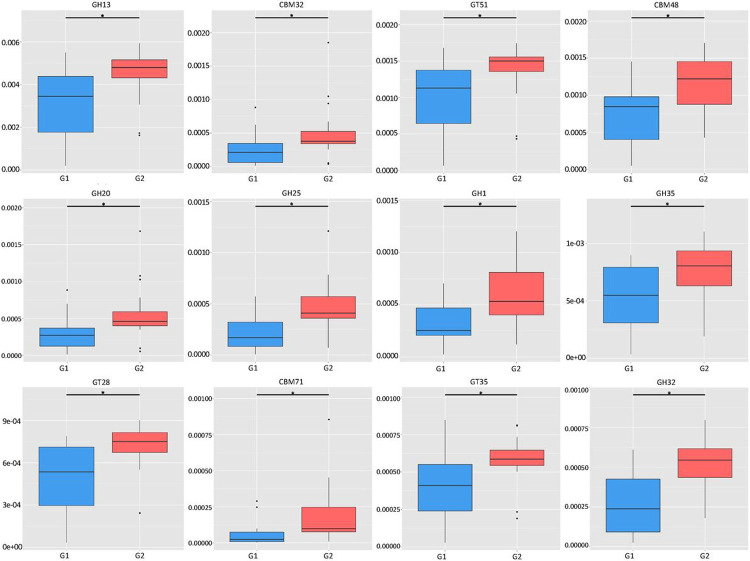
Variation of abundance of carbohydrate-active enzymes in samples with different *S. cristatus-P. gingivalis* ratios. Box-plots represent richness and evenness of the top 12 genes encoding enzymes involved in bacterial metabolism. The y-axis of boxplots represents gene abundance. An asterisk refers significant difference in abundance of the enzymatic genes between two groups. Abbreviations: GH, Glycoside Hydrolase Family; CBM, Carbohydrate-Binding Module Family; and GT, Glycosyl Transferase Family.

In the light of the observations on differential abundance of functional genes, we then investigated the molecular interaction and reaction networks using KEGG pathway databases and compared them between the samples with low or high ratios of *S. cristatus-P. gingivalis*. The results revealed that genes involved in human diseases-associated antimicrobial drug resistance were enriched in the samples with the low ratios (*p* = 0.0498) (Fig. 8). While genes encoding enzymes essential for carbohydrate metabolism were more abundant in the samples with high ratios (*p* = 0.0324), which was in agreement with the analyses on the CAZy database (Fig. 9). The functional profile of the oral microbiome showed significant variations in abundance of genes involved in carbohydrate processing between the groups with different *S. cristatus-P. gingivalis* ratios, including a reduction in some families of glycoside hydrolase, carbohydrate-binding module, and glycosyl transferase in the samples with low ratios of *S. cristatus-P. gingivalis*. Moreover, genes involved in metabolic pathways of amino acid (*p* = 0.040), energy (*p* = 0.0463), amino acid metabolism (*p* = 0.0403) and nucleotide (*p* = 0.0296) as well as glycan biosynthesis and metabolism (*p* = 0.0495) were significantly abundant in the group with high ratios (Fig. 8). However, there is no significant difference in pathways environmental information processing (signal transduction and membrane transport), genetic information processing (translation), metabolism of cofactors and vitamins, cofactors vitamins. These observations suggest a potential association of *S. cristatus-P. gingivalis* ratios with functional variation in the oral microbiome.

## Discussion

A series of studies in our laboratory has established a specific role of *S. cristatus* on violence potential of *P. gingivalis* and on formation and composition of oral microbial biofilms using qPCR (9, 10, 14, 15, 33, 34). Although negative correlation of *S. cristatus-P. gingivalis* has been identified in dental plaque samples derived from periodontitis patients, it is currently not clear how this relationship influences microbial communities as a whole. The study presented here provides a first glance on intraspecies interactions associated with different ratios of *S. cristatus-P. gingivalis* using metagenomic shotgun sequencing. Besides identification of over 1400 microbial taxa at the species level in plaque samples from 30 periodontitis patients, this approach revealed significant differences in abundance, diversity, and functions between two groups of dental plaque samples with high or low *S. cristatus-P. gingivalis* ratios, respectively. Earlier studies by Dewhirst *et al.* (35, 36) estimated, using 16S rRNA sequencing, there may be about 600 common taxa in the human oral microbiome. With powerful shotgun metagenomic sequencing here, we successfully identified over 1400 microbial species, including ones at a very low abundance, therefore, enabling future investigation of potential roles of low prevalent taxa in oral health and diseases.

Our study also revealed significant difference in abundances of common bacterial species between two groups with different *S. cristatus-P. gingivalis* ratios. Well-known periodontitis associated bacteria, including *T. denticola, T. forsythia*, and *F. alocis*, were much abundant in the samples with low *S. cristatus* and *P. gingivalis* ratios. Along with low *S. cristatus* and *P. gingivalis* ratios, we found a close correlation of abundance among *P. gingivalis, T. forsythia, T. denticola*, and *D. oralis* in the samples tested. The correlation is likely due to coaggregation between/among these bacterial species. The molecular mechanisms of coaggregation between *P. gingivalis* and *T. denticola* have been found to include interaction of *P. gingivalis* fimbrial protein and *T. denticola* dentilisin (37). Moreover, *T. denticola* dentilisin is also known responsible for coaggregation between *T. denticola* and *T. forsythia* (38). *P. gingivalis* Hgp44 may also involve in *P. gingivalis* adhesion to *T. denticola*, as a truncated Hgp446 fragment reduced the coaggregation of *P. gingivalis* and *T. denticola* (39). A similar observation reported by Zhu *et al.* indicated an essential role of *P. gingivalis*gingipains in synergistic polymicrobial biofilm formation of *P. gingivalis* and *T. denticola* (40). Unlike well studied pathogenicity of *P. gingivalis, T. denticola*, and *T. forsythia*, the role of *D. oralis* in pathogenesis of periodontitis is unclear. *D. oralis*, which was first isolated and purified from subgingival plaque samples from a periodontitis patient (41), is defined as a non-motile Gram negative and rod-shaped bacterium. Pathogenicity of *D. oralis* may rely on its ability to stimulate production of IL-1β, IFN-α, IFN-γ, MCP-1, IL-6, IL-8, and IL-1 by oral keratinocyte cells (41), suggesting that the organism plays a role in periodontal inflammation. Although there is no evidence that *D. oralis* physically interacts with *P. gingivalis, T. forsythia*, and/or *T. denticola*, our discovery of a cluster abundance of these four bacteria and increased levels of all four species in high ratios of *S. cristatus-P. gingivalis* indicates that *D. oralis* may cooperate with these bacteria as a core microbiota responsible for etiology and progression of periodontitis.

The shotgun metagenomic sequencing also allowed us to identify sixty-nine antibiotic resistance genes in dental plaques from the 30 periodontitis patients. Among these genes, forty-eight were found in both groups, and twenty-one only in the group with low *S. cristatus-P. gingivalis* ratios. Additionally, four out of five most abundant antibiotic resistance genes, *tem-116, cat*I, *aph3*-IIa, and IIIa, were predominantly detected in the group with low *S. cristatus-P. gingivalis* ratios compared to its counterpart. The observation of more diverse and abundant genes in the group with the low ratios may result from the diversity of microorganisms in the samples in this group. Another explanation is that the low ratio of *S. cristatus-P. gingivalis* associated microbiome create an environment facilitating the transmission of resistance genes. Horizontal gene transfer is known as a major pathway for spreading antibiotic resistance gene, e.g., an outbreak of multidrug-resistant nosocomial pathogens caused by a transferable plasmid encoding SHV-12 extended-spectrum β-lactamase (TEM-116) (42). Our discovery of more diverse and abundance antibiotic resistance genes in the low ratio group implies the important role of *S. cristatus-P. gingivalis* ratios in controlling pathogenicity of the oral microbial communities.

As reported in several studies of 16S rRNA sequencing (43, 44), abundance and diversity of several bacterial genera shifted during periodontal health and disease transition. The dental plaque samples in this study were collected from patients with stage II and III periodontitis. Despite no significant difference in gender, age, tooth numbers, BOP (bleeding on probing) scores, and PI (plaque index) between two tested groups that were designated based on their *S. cristatus-P. gingivalis* ratios, significant differences in abundance and diversity of bacterial species as well as functional pathways between the two groups exist. We postulate that *S. cristatus-P. gingivalis* ratios associated increase in abundance and diversity of periodontitis- associated bacteria and antibiotic resistance genes accelerate progression of periodontitis and reduce response to periodontitis treatment. This notion is supported by our previous observations that the ratio of *S. cristatus-P. gingivalis* was significantly higher in Caucasians Americans (CAs) than in Africa Americans (AAs) and that better gains in clinical attachment levels were observed in CA periodontitis patients compared to those found in AAs after nonsurgical periodontal treatment (14, 45).

In conclusion, our work highlights the role of *S. cristatus-P. gingivalis* ratios in virulence potentials of the oral microbiome. Microbial communities with low *S. cristatus-P. gingivalis* ratios had an elevated level of several well-studied periodontitis-associated bacteria, decreased levels of streptococcus and actinomyces species, and much diversified microbial profiles and antibiotic resistance genes. Differential diversity and abundance of the oral microbiome are likely regulated by core bacteria in microbiota, including the ratios of *S. cristatus-P. gingivalis*, which may lead to functional changes of the oral microbiome. Therefore, approaches to enhance *S. cristatus-P. gingivalis* ratio will be attractive for rebuilding and maintaining healthy oral microbiome.

## Figures and Tables

**Table 1:** Characteristics of the study cohort.

	*S. cristatus-P. gingivalis* ratio		
Characteristics	< 1	> 100	*P* – value
Gender (Male/Female)	6/8	12/4	0.078
Age (year; Mean ± SD)	54.6 ± 13.3	64.1 ± 12.2	0.052
BOP (%, Mean ± SD) ^a^	52.9 ± 22.3	44.6 ± 31.0	0.413
PI (%, Mean ± SD) ^b^	69.1 ± 27.2	69.7 ± 31.5	0.958
Tooth number (Mean ± SD) ^c^	26.7 ± 1.9	25.9 ± 1.8	0.232

a BOP: Bleeding on probing

b PI: Modified O'Leary plaque index

c Tooth number is based on a total of 32.

**Table 2:** Differential abundance of antibiotic resistance genes between groups with low or high S. cristatus-P. gingivalis ratios

Antibiotic resistance genes	Gene function	Total of gene copies		Fold changes	p- value
		G1	G2	G1/G2	
*tem-116*	β-lactamase β-lactam antibiotics	24527.77	42.29	580.03	0.045
*catI*	Chloramphenicol acetyltransferase chloramphenicol	16825.01	26.61	632.28	0.045
*aph3*-IIa	Aminoglycoside phosphotransferase	8841.63	10.39	851.09	0.045
*aph3*-IIIa	Aminoglycoside-3'-Phosphotransferase kanamycin	860.43	2.22	388.38	0.045
*van*T_gene_in_*van*G_cluster	Resistance to vancomycin and teicoplanin type antibiotics	114.94	149.04	0.77	0.164
*erm*F	Member of RNA methyltransferase family	26.46	45.72	0.58	0.349
*tet*Q	Tetracycline-resistant ribosomal protection protein	25.05	33.31	0.75	0.586
*pat*B	Associated with fluoroquinolone resistance	14.38	44.64	0.32	0.023
*pat*A		12.42	45.82	0.27	0.014
*Escherichia coli*_EF-Tu_mutants_conferring_resistance_to_Pulvomycin	Resistance to Pulvomycin	7.54	14.66	0.51	0.399
ErmB	A member of RNA methyltransferase family	2.86	15.25	0.19	0.123
*van*W_gene_in_*van*I_cluster	Resistance to vancomycin and teicoplanin type antibiotics	31.52	50.32	0.63	0.031
*van*Y_gene_in_*van*M_cluster		25.54	42.52	0.60	0.073
*van*Y_gene_in_*van*G_cluster	Tetracycline-resistant ribosomal protection protein	5.58	23.15	0.24	0.010
*tet*M		8.40	24.48	0.34	0.026
*cfx*A2	Beta-lactamase	17.17	26.61	0.65	0.271
*van*W_gene_in_*van*B_cluster	Resistance to vancomycin and teicoplanin type antibiotics	21.36	1.59	13.45	0.001
*pgp*B	Phosphatidylglycerophosphatase	22.91	0.05	441.68	0.000
*qac*G	Resistance to benzalkonium chloride and ethidium bromide	9.82	23.83	0.41	0.039
*Haemophilus_influenzae*_PBP3_conferring_resistance_to_beta-lactam_antibiotics	Resistance to beta-lactam antibiotics	2.92	6.37	0.46	0.320
nimI	Nitroimidazole reductase	6.83	10.44	0.65	0.425
*van*W_gene_in_*van*G_cluster	Resistance to vancomycin and teicoplanin type antibiotics	7.36	6.04	1.22	0.705
*tet*W	A protein binds to the 30S ribosomal subunit	1.03	5.68	0.18	0.061
*van*Y_gene_in_*van*B_cluster	Resistance to vancomycin and teicoplanin type antibiotics	7.78	11.10	0.70	0.270
*mde*A	Multidrug efflux pump	2.02	7.44	0.27	0.077
*tet*32	Tetracycline-resistant ribosomal protection protein	5.87	6.40	0.92	0.807
*van*Y_gene_in_*van*F_cluster	Resistance to vancomycin and teicoplanin type antibiotics	12.66	10.04	1.26	0.403
*Klebsiella pneumoniae_kpn*H	Pumping of antibiotic out of a cell to confer resistance	0.76	3.29	0.23	0.104
*nim*J	Deactivation of nitroimidazole antibiotics	1.51	6.16	0.25	0.007
*ade*F	Antibiotic efflux	4.03	3.92	1.03	0.941
